# Combination Antifungal Therapy in the Treatment of *Scedosporium apiospermum* Central Nervous System Infections

**DOI:** 10.1155/2013/589490

**Published:** 2013-04-29

**Authors:** Andrés F. Henao-Martínez, José R. Castillo-Mancilla, Michelle A. Barron, Aran Cunningham Nichol

**Affiliations:** Division of Infectious Diseases, University of Colorado Denver, 12700 E. 19th Avenue, Mail Stop B168, Aurora, CO 80045, USA

## Abstract

Treatment of *Scedosporium apiospermum* central nervous system (CNS) infection typically consists of an azole in combination with surgical debridement. This approach requires prolonged treatment and carries a high associated mortality. We present two cases of the successful treatment of *S. apiospermum* CNS infections with the combination of voriconazole and terbinafine.

## 1. Case Reports

### 1.1. Case  1

**Figure 1:**
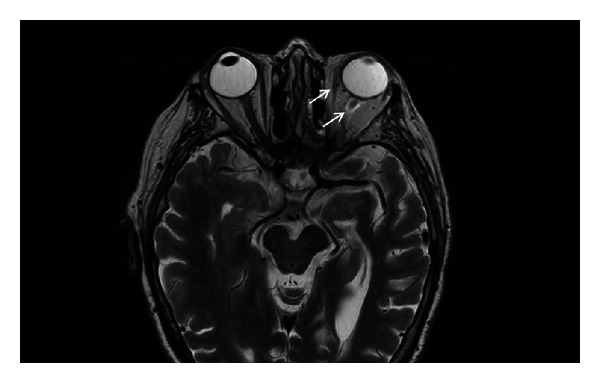
Orbital MRI. Orbital MRI revealed mildly elevated signal within the intra-orbital segment of the left optic nerve, mild inflammatory change surrounding the optic nerve sheath and very mild asymmetric prominence of the left extra-ocular muscles concerning for myositis (arrows).

An 81-year-old Hispanic woman with a past medical history significant for hypertension, diabetes mellitus, hypothyroidism, and chronic kidney disease presented with symptoms of sinusitis, including facial pain, nasal congestion, and rhinorrhea. The sinusitis was later complicated by the development of left orbital cellulitis. The patient underwent a functional endoscopic sinus surgery (FESS) without evidence of gross infection and was treated with oral levofloxacin. Three weeks later, her orbital cellulitis resolved; however, she developed nausea, vomiting, headaches, left vision loss, diplopia, and local pain. Her eye exam was significant for intact extraocular movements, no nystagmus, and a positive afferent pupillary defect. No periorbital edema or erythema was observed. Visual acuity for her right eye was 20/60 and for her left eye was 20/100. Her basic laboratory results were normal. She was diagnosed with a left orbital complex syndrome with optic neuropathy and underwent another FESS with sinusotomy and debridement. The patient was briefly placed on intravenous (IV) steroids, and she was discharged home on an oral prednisone taper and levofloxacin. However, the patient was readmitted 10 days later with worsening vision loss on the left; she was then only able to perceive light. The intraoperative cultures from her last FESS grew a moderate amount of mold, which was subsequently identified as *Scedosporium apiospermum *based on its characteristic macroscopic morphology and conidiation. Susceptibility testing of the isolate revealed a voriconazole MIC of 0.5 ug/mL. An orbital MRI revealed mildly elevated signal within the intraorbital segment of the left optic nerve, mild inflammatory changes surrounding the optic nerve sheath complex and the orbital apex, and very mild asymmetric prominence of the left extraocular muscles concerning for myositis (Figure 1). The patient was placed on IV voriconazole at 6 mg/kg every 12 hours for a presumed fungal sinusitis, myositis, and possibly sinus fungal osteomyelitis. After approximately 30 days of IV voriconazole therapy, the patient was transitioned to oral voriconazole and had obtained a voriconazole trough level of 2.06 ug/mL a month later. Two months after antifungal therapy was initiated, the patient presented with worsening headache, nausea, vomiting, and anorexia, and a repeat orbital MRI showed new mild left proptosis, enhancing soft tissue at the orbital apex extending along the cavernous sinus and under both frontal lobes along the dura, and slightly increased mucosal thickening throughout the sinuses with moderate to severe involvement of the left sphenoid and right maxillary sinus. The patient was evaluated for possible surgical debridement; however, due to the complexity of the disease location, it was decided to proceed with medical management only. Due to the rapid progression of her disease, renally dosed oral terbinafine was added at a dose of 250 mg daily. After 4 months of combination therapy, repeat orbital MRI showed stable inflammatory changes without progression. The patient's clinical status eight months later is stable with no further CNS symptoms and good tolerance of her antifungal regimen.

### 1.2. Case  2

**Figure 2:**
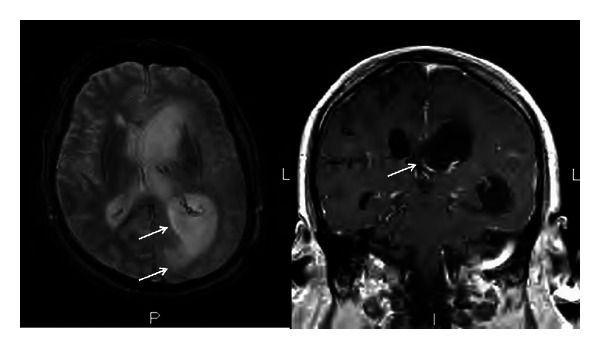
Brain MRI. Ventriculomegaly, increased halo surrounding the lateral ventricles, and debris within the ventricles with restricted diffusion and ependymal and subependymal enhancement (arrows).

**Figure 3:**
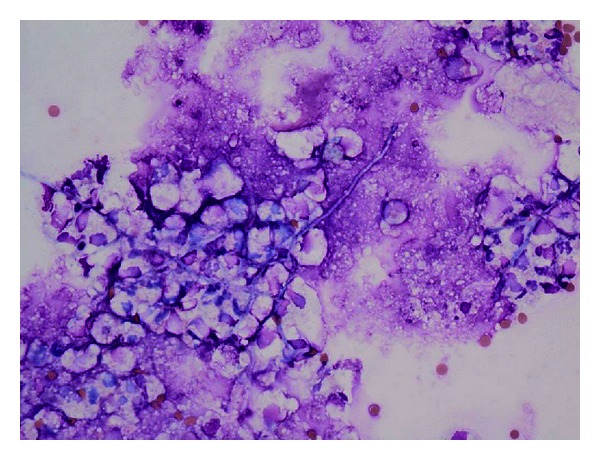
H&E stain of the Brain Biopsy. Clusters of amorphous linear structures with a morphology simulating fungal hyphae.

A 64-year-old White man with a previous diagnosis of eosinophilic pneumonia, which had been treated with high-dose steroids, cyclophosphamide, and mycophenolic acid, presented with subacute symptoms of headaches, fevers, aphasia, and ataxia. He was diagnosed with meningitis, cerebritis, and ventriculitis based on an elevated cell count in his cerebral spinal fluid (CSF) and consistent imaging; however, the etiology was unclear. He was treated with antibacterial therapy, including a 10-day course of vancomycin, cefepime, and ampicillin, with partial resolution of his symptoms. He had a negative infectious workup including a negative brain biopsy, as well as, a normal cerebral angiogram. A CT scan of the head demonstrated hydrocephalus, and he had an external ventricular drain placed. A month later, his mental status worsened. At that time, his vital signs were normal and his neurologic exam was notable for mild confusion. Cranial nerves II through XII were intact with the exception of slow saccades, but no nystagmus was present. Laboratory studies were remarkable for a slightly elevated white blood cell count (WBC) of 12.2 × 10^3^/uL (normal range: 4.5–11.0 × 10^3^/uL) and a decreased hematocrit (Hct) of 29.1% (normal range: 42–54%). A repeat evaluation of his CSF showed 326 WBC (normal range: 0–5 × 10^6^/L) with 83% neutrophils (normal range: 0–6%). His CSF protein was 113 mg/dL (normal range: 15–45 mg/dL) and glucose was 54 mg/dL (normal range: 40–80 mg/dL). Gram stain and bacterial cultures were negative. An extensive infectious workup was negative including, CSF AFB smear and culture, serum and CSF cryptococcal antigen, *Toxoplasma gondii* antibody, Parvovirus B19 antibody, CSF VDRL, CSF and serum PCR for HHV-6, EBV, HSV-1/2, CMV, WNV, enterovirus, and JC virus. An MRI of his brain revealed ventriculomegaly, an increased halo surrounding the lateral ventricles, and debris within the ventricles with restricted diffusion and ependymal and subependymal enhancement (Figure 2). A repeat brain biopsy showed fungal-like elements (Figure 3). His CSF fungal cultures subsequently grew *Scedosporium apiospermum*. He was then started on oral voriconazole 600 mg twice daily and terbinafine 250 mg twice daily due to the severity of the infection. Two weeks later the voriconazole trough level was 2.58 ug/mL. His mental status improved substantially. Three months later, repeat CSF studies showed 3 WBC (normal range: 0–5 × 10^6^/L) with 3% neutrophils (normal range: 0–6%). His CSF protein was 42 mg/dL (normal range: 15–45 mg/dL) and glucose was 60 mg/dL (normal range: 40–80 mg/dL). Repeat CSF fungal cultures were negative. In light of his dramatic improvement, terbinafine was stopped and he continued on voriconazole only with regular monitoring of his drug level. Unfortunately, two months later the patient presented again with fevers and confusion, and his CSF cultures grew *Scedosporium apiospermum*. His clinical course was then complicated by influenza pneumonia, pulmonary embolism, ventricular drain malfunction, and renal failure. The patient failed to improve and ultimately was discharged to hospice and died one month later. 

## 2. Discussion

*Scedosporium apiospermum*, the anamorphic (asexual state) of *Pseudallescheria boydii,* is a ubiquitous organism with a worldwide distribution [1, 2]. This organism is most commonly associated with localized cutaneous and subcutaneous infections of the lower extremities after traumatic penetration with contaminated material (Madura foot) but can also cause disease at other sites such as the lung, brain, bone, eye, sinuses and joints [3–5]. Invasive and disseminated disease presenting with severe pneumonia, CNS infection, and endocarditis has been described in immunocompromised hosts, where the respiratory tract has been identified as the main major portal of entry [6–8]. In a review of 23 *S. apiospermum* infections in solid organ transplant recipients between 1976 and 1999, 8 patients developed dissemination, 11 had invasive lung disease, and 11 had CNS involvement, including 10 patients with brain abscesses [9]. In another series, almost 60% of 39 culture-proven cases of *S. apiospermum* infections of the CNS presented with an underlying immune impairment (immunosuppressive drugs, antineoplastic chemotherapy, or with a medical immune deficiency) [10]. In an Australian retrospective review of *Scedosporium *infections, *S. apiospermum *demonstrated invasiveness in 6% of cases, whereas *S. prolificans *was invasive in 36% of cases [11]. Regarding antifungal susceptibility, *in vitro* data suggest that the two antifungals with the best activity against *S. apiospermum *are voriconazole and micafungin [12]. Voriconazole has shown clinical efficacy and good tolerance in the management of *Scedosporium* spp. infections [13]. Measurements of voriconazole levels have been shown to be an effective method to improve efficacy and safety in this setting [14, 15]. Although *in vitro* data for terbinafine have shown high MICs against *Scedosporium spp.*; MICs in the literature are variable [16, 17], activity has been noted for some of the isolates [18, 19], and *in vitro* synergy between azoles and terbinafine has been reported for *S. prolificans* [17]. Likewise, terbinafine and voriconazole have been used successfully as combination therapy in the treatment of invasive *S. apiospermum *[20–23] and *S. prolificans* [24–26]. There is also a report of successful treatment of *S. apiospermum* with voriconazole in combination with caspofungin [27] or with posaconazole alone [28]. 

Predisposing factors for progressive fungal CNS infection in the first case we presented included advanced age, previous antibiotic and steroid exposure, the presence of diabetes mellitus and recent sinus instrumentation. As an alternative to terbinafine, an IV echinocandin could have been considered in combination with oral voriconazole in this case, but the likelihood of a prolonged, and possibly lifelong, treatment course made IV administration less feasible. The second patient had intense immunosuppressive therapy as his main predisposing factor for progressive fungal CNS infection. He clearly had a notable improvement with normalization of CSF cell counts and CSF sterilization while on combination antifungal therapy; however, due to his multiple comorbidities and a prolonged hospitalization, the infection relapsed and he died. Although successful reports of this antifungal combination have been described in the treatment of *S. prolificans* [24–26, 29], to our knowledge this is only the third case report of the successful use of the combination of voriconazole and terbinafine in the treatment of invasive CNS *Scedosporium apiospermum* infection. This treatment combination should be considered in cases of severe CNS infection with *Scedosporium apiospermum,* where surgical options are limited, and/or there has been a suboptimal response to voriconazole monotherapy.
